# The effect of placental location identified before delivery on birthweight discordance among diamniotic-dichorionic twin pregnancies: a three-year retrospective cohort study

**DOI:** 10.1038/s41598-019-48667-3

**Published:** 2019-08-20

**Authors:** Dongxin Lin, Shuzhen Wu, Dazhi Fan, Pengsheng Li, Gengdong Chen, Huiting Ma, Shaoxin Ye, Jiaming Rao, Huishan Zhang, Ting Chen, Meng Zeng, Yan Liu, Xiaoling Guo, Zhengping Liu

**Affiliations:** 1 Foshan Institute of Fetal Medicine, Southern Medical University Affiliated Maternal & Child Health Hospital of Foshan, Foshan, Guangdong 528000 China; 2 Department of Obstetrics, Southern Medical University Affiliated Maternal & Child Health Hospital of Foshan, Foshan, Guangdong 528000 China

**Keywords:** Outcomes research, Risk factors

## Abstract

This retrospective cohort study aimed to investigate the effect of placental location on birthweight discordance among diamniotic-dichorionic twin pregnancies. Medical records and sonographic reports of 978 diamniotic-dichorionic twin pregnancies delivered at Foshan Maternal and Fetal Health Hospital were reviewed. Pregnancies with congenital malformation, intrauterine death or placenta previa were excluded. The placental location for each twin was determined by last sonographic examination before delivery, and the pregnancies were grouped by different versus same placental location in each pregnancy. Maternal and fetal characteristics were summarized. The primary outcome of interest was birthweight discordance (BWD) ≥20%, and secondary outcomes included small for gestational age (SGA) as a binary outcome and mean value and absolute difference in birthweight as continuous outcomes. Student’s *t* test and the chi-square test were used for univariate analyses, while multivariate regressions were used to adjust for confounders. General estimated equation (GEE) models were used to address the correlation between fetuses when assessing SGA. A total of 866 eligible subjects were included in the analysis. In total, 460 pregnancies had placentas with different locations, and 406 had placentas with same locations. The gestational age at delivery was slightly younger in the same placental location group than in the different placental location group (35.8 ± 0.1 vs. 36.1 ± 0.1 weeks, *P* = 0.067). Other maternal and fetal characteristics were comparable between the two study groups. There was no significant difference in BWD ≥20% (aOR = 1.06; 95% CI: 0.71–1.59) or SGA (aOR = 1.32; 95% CI: 0.76–2.28) between the same and different placental location groups. Neither the mean value nor the absolute difference in birth weight was associated with placental location combination (*P* = 0.478 and *P* = 0.162, respectively). In conclusion, discordant birthweight is not affected by same location of diamniotic-dichorionic placentas.

## Introduction

In recent years, the number of twin pregnancies has been steadily growing with the increasing use of assisted reproductive technology (ART)^1^. Twin gestations are associated with a series of adverse outcomes such as preterm birth, congenital anomalies, fetal loss, fetal growth restriction and neonatal death^2–5^. Accumulating evidence has shown that growth discordance with small for gestational age (SGA) among twin gestations is related to increased perinatal morbidity and mortality, as well as long-term adverse neuro-developmental outcomes^6,7^.

Identifying predictors of fetal growth is of great benefit in patients counseling and antepartum management. Previous studies have investigated predictors such as maternal and fetal factors, including maternal weight gain during pregnancy, maternal complications, methods of conception, chorionicity and neonatal sex^5,8,9^. In addition, because the placenta plays a key role in mediating the growth and development of twin fetuses, considerable efforts have been made to evaluate the effect of placental characteristics on fetal growth. Abnormal cord insertion (velamentous and marginal) and placental volume were demonstrated to be associated with increased risks of growth retardation and discordance^10^. Venovenous anastomoses were proven to be associated with birth weight discordance in monochorionic-diamniotic twin pregnancies^11^.

Placental location was reported to be related to birthweight among singleton pregnancies^12–14^, mainly due to decreased blood supply to lateral and fundal placentas. Evidence for the role of placental location, nevertheless, among diamniotic-dichorionic twins is sparse. The mechanism of discordant growth among diamniotic-dichorionic twins (DC) is not clear. We hypothesized that, when both placentas were covering on the same area of the uterus wall, there exists an intertwin competition for blood supply, which might subsequently affect the fetal growth. Belogolovkin *et al*.^15^ retrospectively reviewed the medical records of a medium-sized population (292 pairs of diamniotic-dichorionic twins) and found no correlation between birth weight and placental location. This result, however, may be of limitedly utility because of the inclusion criteria for eligible participants. To the best of our knowledge, no other results from a different center have been reported.

In this regard, we sought to investigate the association between placental location and birthweight discordance among diamniotic-dichorionic twins in a large population.

## Results

**Figure 1:**
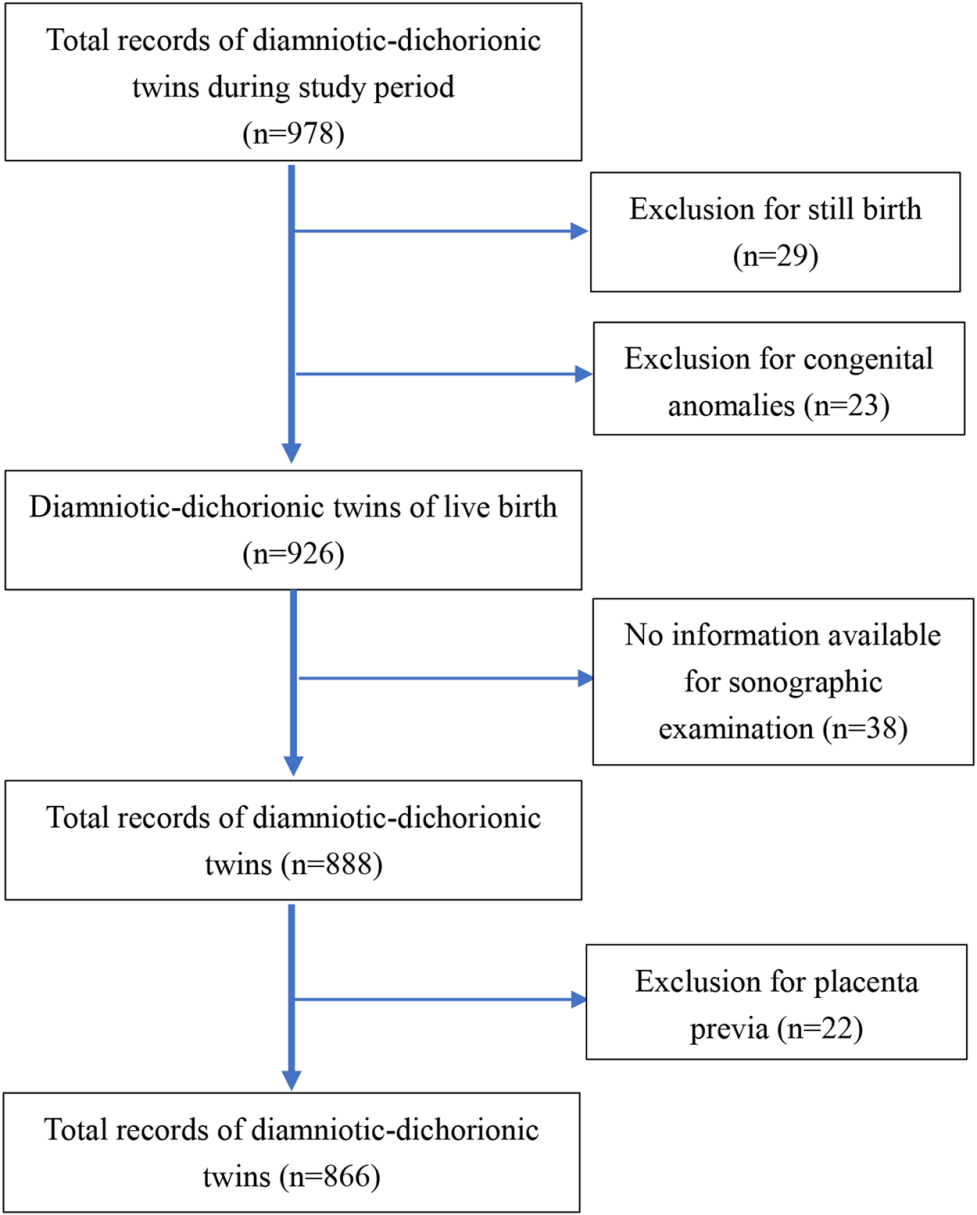
Flowchart of selection of eligible subjects.

The flowchart of the study group selection is represented in Fig. 1. There were 978 diamniotic-dichorionic twin pregnancies admitted to our tertiary referral hospital during the study period. We excluded non-live twin births (stillbirth and medical or spontaneous abortion) (2.97%) and births with congenital anomalies (2.35%). After the medical records were systematically reviewed, thirty-eight twin pairs without available sonographic information were excluded (3.89%). Additionally, twenty-two cases with placenta previa (2.25%) were also excluded, leaving a total of 866 diamniotic-dichorionic twin pregnancies for analysis.

Sonographic examinations were performed on the included participants at a median gestational age of 35.9 weeks (range: 26.8–38.2 weeks). The maternal-fetal characteristics by placental location are shown in Table 1. Among eligible subjects, 460 had different placental locations, while the remaining 406 had same placental locations. In the same placental location group (n = 406), 202 and 178 pregnancies had both anterior placentas or both posterior placentas, respectively; only a few involved one or two placentas (n = 26) located on the lateral or fundal uterine wall. In different placental location group (n = 460), 388 pregnancies had one anterior and one posterior placenta; only a small proportion had one left and one right placenta (n = 41) or other combinations (n = 31). Most of the included pregnancies (n = 680) were conceived by assisted reproductive technology (ART) including 663 cases of vitro fertilization and embryo transfer (IVF-ET), 14 of artificial insemination (AI) and 3 of Intracytoplasmic sperm injection (ICSI). The gestational age at delivery was slightly younger in the same placental location group than in the different placental location group (35.8 ± 0.1 vs. 36.1 ± 0.1, *P* = 0.067). There were no differences between the two study groups in maternal-fetal characteristics, including maternal age, use of ART, history of pregnancy loss, maternal complications, abnormal cord insertion and neonatal sex.

**Table 1:** Maternal and fetal characteristics by placental locations.

Characteristics	Different placental locations (N = 460)	Same placental locations (N = 406)	*P*-value
Maternal age ≥35	117 (25.4)	104 (25.6)	0.951
Gestational age at delivery	36.1 ± 0.1	35.8 ± 0.1	0.067
Nulliparity	263 (64.8)	322 (70.0)	0.101
Use of ART	363 (78.9)	317 (78.1)	0.765
History of spontaneous pregnancy loss	77 (16.4)	82 (20.2)	0.190
Gestational hypertensive disorder	51 (11.1)	41 (10.1)	0.638
Pre-pregnancy or gestational diabetes	102 (23.2)	88 (22.3)	0.859
ICP	9 (2.0)	7 (1.7)	1.000
Chorioamnionitis	7 (1.5)	7 (1.7)	1.000
Abnormal cord insertion	13 (2.8)	7 (1.7)	0.366
Opposite neonatal sex			0.236
Male-male	145 (31.52)	138 (33.99)	
Female-male	214 (46.52)	166 (40.89)	
Female-female	101 (21.96)	102 (25.12)	
Combination of placental locations	Anterior-posterior: 388 (84.3) Other combinations*: 72 (15.6)	Anterior-anterior: 202 (49.8) Posterior-posterior: 178 (43.8) Other combinations*: 26 (6.4)	—

ART, assisted reproductive technology; ICP, intrahepatic cholestasis of pregnancy. *Placental location combination involved fundal or lateral placentas.

The overall prevalence of birth weight discordance ≥20% and SGA pregnancy was 10.7% and 6.8%, respectively. Neonatal outcomes by study groups in the univariate analysis are shown in Table 2. There were no differences in the incidence of birth weight discordance ≥20% (10.65% vs. 10.84%, *P* = 0.930) or SGA pregnancy (6.30% vs. 7.38%, *P* = 0.527) between the two study groups. Regarding continuous outcomes, the mean birth weight was slightly higher in the different placental location group (2.35 ± 0.01 vs. 2.30 ± 0.02, *P* = 0.089) and the absolute difference in birthweight was significantly larger in the different placental location group (0.26 ± 0.01 vs. 0.23 ± 0.01, *P* = 0.045).

**Table 2:** Neonatal outcomes by placental location in univariate analysis.

Outcomes	Different placental locations (N = 460)	Same placental locations (N = 406)	*P*-value
Birth weight discordance ≥20%, n	49 (10.65%)	44 (10.84%)	0.930
SGA in twin A, n	25 (5.43%)	28 (6.90%)	0.370
SGA in twin B, n	24 (5.22%)	26 (6.40%)	0.455
SGA pregnancy (in at least one twin), n	29 (6.30%)	30 (7.38%)	0.527
Mean birth weight of both twin fetus, kg	2.348 ± 0.019	2.301 ± 0.020	0.089
Absolute birth weight difference, kg	0.262 ± 0.010	0.234 ± 0.010	0.045

Note: SGA, small for gestational age.

After applying multivariate logistic models to adjust for potential confounders, no correlations were found between placental location and the incidence of birth weight discordance ≥20% (aOR: 1.06; 95% CI: 0.71–1.59) or SGA (aOR: 1.32; 95% CI: 0.76–2.28) (Table 2). For continuous outcomes, neither the mean value (β = −0.012, *P* = 0.478) nor the absolute difference (β = −0.019, *P* = 0.162) in birth weight was correlated with placental location (Table 3).

**Table 3:** The relationship between neonatal outcomes and placental location in multivariate adjusted models with different placental locations group as reference.

Outcomes	aOR	95% CI	*P*-value
Birth weight discordance ≥20%*	1.06	0.71–1.59	0.777
SGA fetus^#^	1.32	0.76–2.28	0.321
**Outcomes**	**β**	**95% CI**	***P***-**value**
Mean birth weight of both twin fetus, kg^†^	−0.012	−0.046–0.022	0.478
Absolute birth weight difference, kg^†^	−0.019	−0.046–0.008	0.162

Note: aOR, adjusted odds ratio; CI, confidence interval; SGA, small for gestational age.

*Adjusted for neonatal sex, abnormal cord insertion and pre-gestational or gestational diabetes mellitus.

^#^GEE model adjusted for neonatal sex, abnormal cord insertion and pre-gestational or gestational diabetes mellitus.

^†^Adjusted for gestational age at delivery, neonatal sex, abnormal cord insertion and pre-gestational or gestational diabetes mellitus.

We further compared outcomes between pregnancies that had both anterior placentas, both posterior placentas or one anterior and one posterior placenta. No difference was found in neonatal outcomes between these three groups (see Tables S1 and S2).

## Discussion

This retrospective cohort study was based on sonographic reports of placental location and the medical records on birth weight. No associations were found between placental location and birth weight discordance, SGA or mean value and absolute difference in birth weight.

While the placenta is a unique organ that delivers nutrition to the fetus, it is generally accepted that fetal growth retardation is associated with abnormal placentation, leading to decreased blood supply to the fetus. In singletons, fundal and lateral placentas are reported to be associated with a higher risk of SGA infants^14,16^, which is explained by the decreased blood supply to the placentas. We speculated that among diamniotic-dichorionic twins, separately located placentas are more likely to receive blood supply from different areas of the uterus, while placentas with same locations can receive from only one area; therefore, fetal growth may be limited. Since there is currently no official classification regarding placental location, we classified placental location as anterior, posterior, lateral (right or left) or fundal, as in previous studies^14,16,17^. We grouped cases according to whether the two placentas shared the same uterine wall. A large population (n = 866) of twin pregnancies with all types of placental locations, except placenta previa, were included. However, the results refuted our hypothesis, that was, placentas that shared the same uterine wall did not lead to a higher risk of severe discordant birthweight, or intrauterine growth restriction. This finding is similar to that of a previous study by Belogolovkin *et al*.^15^, although their inclusion criteria may have introduced some selection bias because they included only twin pairs with specific placental types (anterior and posterior placentas). Blickstein *et al*.^18^ reported a similar incidence of SGA and birth weight discordance between dichorionic twins with fused and separate placentas. This finding is in accordance with ours despite the use of different placental location categories. Based on existing evidence, one possible explanation is that a shared uterus wall shared by two placentas can provided the uteroplacental perfusion for both fetuses; otherwise, one or both would migrate towards a site providing better nourishment. As reported by prior studies, abnormal cord insertion has implications for fetal growth^10,19^. We detected trends in both SGA and discordance ≥20% when the pregnancies were complicated with abnormal cord insertion; nevertheless, these trends failed to achieve a customary level of statistical significance (data not shown), which may be due to the low incidence among our study population.

A panel of biomarkers in combination with ultrasound finding and clinical characteristics for the early diagnosis of preeclampsia and fetal growth restriction, could offer an opportunity to optimize maternal and/or perinatal outcomes, which consists of controlling hypertension, preventing seizures, timely delivery and even prophylactic administration of aspirin. On one hand, increasing evidences in the recent years has identified the role of micro-RNA in pregnancies with these placenta-induced complications^20,21^. However, current evidence is still unable to provide a robust panel of biomarkers, partly due to a lack of statistical power and reproducible methods. On the other hand, the implementation of early serum markers in clinical practice has been hampered by their low sensitivity and specificity and their high cost^22^. Notably, previous evidence was predominantly based on singletons, rarely on diamniotic-dichorionic twins. Recently, Biesiada *et al*.^23^ found dysregulation of gene expression related to angiogenesis and growth factors in placentas of diamniotic-dichorionic twins with discordant growth. Because this study excluded intertwin competition for blood supply as a factor for discordant birthweight, we agreed with that a diamniotic-dichorionic twin pregnancy is a good model as both twins develop in the same maternal environment. Future evidence on this model may provide novel insight into placental biomarkers among SGA fetuses.

Since ART procedure is increasingly used and the majority of ART-related multiple gestations are expected to be dizygotic, our results may be of interest to gynecologists and pediatricians for evaluating the risk of fetal growth restriction in diamniotic-dichorionic twins. While the strength of this study is the large population, several limitations should be noted in our study when interpreting results. First, the classification of placental location in our study may lack precision. Therefore, we believe that terms that describe whether both placentas receive blood supply from the same uterine artery, or the distance between the two placentas, may more clearly delineate the relationship between placental location and birthweight discordance. Second, information on placental location was obtained from examinations in the third trimester; thus, we could have missed cases of placental migration. Our results need to be confirmed by evidence with earlier ultrasound examinations. Third, a large proportion of ART-related twin pregnancies were included in our study. It is reasonable to believe that some of the mothers may have had endometriosis, which is reported to affect up to 50% of infertile women and is considered as a risk factor for SGA newborns^24,25^. However, we were unable to obtain this information due to the retrospective nature of this study. Moreover, these limitations may hamper the extension of our conclusions to other circumstances where there are fewer ART-related pregnancies.

In summary, discordant birthweight among diamniotic-dichorionic twins is not affected by the same or different placental locations. Future studies on ultrasound-based placental characteristics related to discordant growth among diamniotic-dichorionic twins are warranted.

## Methods

### Study population and design

This retrospective cohort study was performed by analyzing the medical records and sonographic reports for diamniotic-dichorionic twin pregnancies who referred to and deliverd at Foshan Maternal and Fetal Health Hospital between July 2015 and June 2018. This study was approved by the Human Subjects Committee of the Southern Medical University Affiliated Maternal & Child Health Hospital of Foshan. All the methods in the present study were performed in accordance with the approved guidelines. Informed consent was obtained from all participants. Eligible subjects were identified through a systematic review of medical records and sonographic reports. Pregnancies with viable twins born at a gestational age of ≥26 weeks were included if complete information on pregnancy outcomes and sonographic examination were available; otherwise, pregnancies were excluded if: (1) major congenital malformations or chromosomal anomalies were noted in medical records; (2) twin pregnancies were affected by intrauterine death or were terminated before gestation 26 weeks of gestational age; or (3) twin pregnancies were complicated by any type of placenta previa. Chorionicity was determined at the first sonographic examination (lambda-sign for monochorionicity and T-sign for diamnioticity) and was confirmed by placental pathologic findings after birth. The gestational age was calculated based on the last menstrual period and confirmed by sonography in the first trimester, if available. Maternal and fetal medical records were reviewed for the following characteristics: maternal age, mode of conception method (spontaneous conception vs. ART), last menstrual period, nulliparity, history of spontaneous fetal loss, presence of maternal complications (gestational hypertensive disorder, preexisting or gestational diabetes, intrahepatic cholestasis of pregnancy and chorioamnionitis) and birth weight. ART included *in vitro* fertilization and embryo transfer (IVF-ET), artificial insemination (AI) and intracytoplasmic sperm injection (ICSI). The presence of abnormal cord insertion (including velamentous and marginal cord insertion) was also recorded upon placental gross examinations. Placental location for each twin was determined in the last transabdominal sonographic examination before delivery and was recorded as anterior, posterior, left, right or fundal placenta when the placenta predominantly (≥80% of the placental area) covered the corresponding position of uterine wall. When a placenta located on one position (<80% of the placental area) but entered another (>20% of the placental area), combination terms were recorded. For example, if “anterior” and “right” were simultaneously indicated, the placental location was recorded as an anterior-right placenta. In this study, twin pregnancies were grouped by same or different placental location group according to whether the placental locations overlapped. Typically, same placental location was defined as both placentas clearly were located on the same uterine wall, such as anterior-anterior, posterior-posterior, left-left placentas or right-right. In addition, pregnancies were placed in the same placental location group when the combination term of one placenta overlapped the location of the other. For example, a pregnancy with an anterior-right placenta and an anterior placenta was placed in the same placental location group. Otherwise, pregnancies with differently located placentas were placed in the different placental location group.

### Outcomes of interest

The primary outcome of interest was birth weight discordance (BWD) defined as an intrapair difference of 20% or greater. The secondary outcomes were small for gestational age, defined as birthweight was below the 10^th^ percentile for gestation age and sex based on twin birth weight curves in Chinese twins^26,27^, and the mean value and absolute difference in birthweight, which were analyzed as continuous variables. The percentage of birth weight discordance was calculated by dividing the actual birth weight difference by the weight of the larger twin and multiplying by 100.

### Statistical analysis

PASS version 11 was used for sample size calculation, and Stata version 13.1 was used for the statistical analysis. The overall incidence of BWD in the different placental location group was presumed to be 10%. To detect a minimal increase of 7% in the same placental location group, with a statistical power of 80% and a confidence level of 95%, 373 pregnancies had to be included in each group. Data are presented as the mean ± SD or frequency and percentage. Study population characteristics and neonatal outcomes were compared between the two study groups (different placental location vs. same placental location) using a t test or the chi-square test. Multivariate logistic models and linear regression models were utilized to adjust for confounders of binary outcomes and continuous outcomes, respectively. Confounding variables included neonatal sex, abnormal placental insertion and maternal diabetes. Gestational age at delivery was also adjusted for when assessing birthweight as a continuous outcome. For SGA fetuses, the general estimated equation (GEE) was used in order to address the correlation between fetuses in a paired set. The results are represented as odds ratios for binary outcomes or as coefficients for continuous outcomes, with corresponding 95% confidence intervals. All *P*-values were two-sided, at a significance level of 0.05.

## Supplementary information

Table S1, Table S2
