# Supplementary material for: The effect of placental location identified before delivery on birthweight discordance among diamniotic-dichorionic twin pregnancies: a three-year retrospective cohort study
Source: Sci Rep. 2019 Aug 20;9:12099. doi: 10.1038/s41598-019-48667-3 (PMC6702179; doi:10.1038/s41598-019-48667-3)
Supplement: Supplementary file 1 — Table S1, Table S2 [file 41598_2019_48667_MOESM1_ESM.pdf]

Title page

Title: The effect of placental location identified before delivery on birthweight discordance among diamniotic-dichorionic twin pregnancies: a three-year retrospective cohort study

Dongxin Lin, Shuzhen Wu, Dazhi Fan, Pengsheng Li, Gengdong Chen, Huiting Ma, Shaoxin Ye, Jiaming Rao, Huishan Zhang, Ting Chen, Meng Zeng, Yan Liu, Xiaoling Guo, Zhengping Liu

Outcomes	Posterior-posterior (n=178)	Anterior-anterior (n=202)	Anterior-posterior (n=388)	*P*-value
BWD ≥ 20%, n	22 (10.89)	21 (11.80)	39 (10.05)	0.817
SGA in any twin, n	14 (6.93)	12 (6.74)	26 (6.70)	0.994
Mean birth weight of both twin fetus, kg	2.292±0.419	2.317±0.396	2.350±0.401	0.251
Absolute birth weight difference, kg	0.252±0.197	0.222±0.196	0.255±0.209	0.154

Table S1. Neonatal outcomes between posterior-posterior, anterior-anterior and anterior-posterior placentas

Binary outcomes	aOR	95% CI	*P*-value
Birth weight discordance ≥ 20%[*]			
Anterior-posterior	Reference	-	-
Posterior-posterior	1.25	0.58-2.69	0.567
Anterior-anterior	0.99	0.45-2.16	0.976
SGA in twin fetus[#]			
Anterior-posterior	Reference	-	-
Posterior-posterior	1.05	0.51-2.18	0.891
Anterior-anterior	1.11	0.56-2.20	0.774
Continuous outcomes	β	95% CI	*P*-value
Mean birth weight of both twin fetus, kg[†]			
Anterior-posterior	Reference	-	-
Posterior-posterior	-0.008	-0.055-0.038	0.724
Anterior-anterior	-0.022	-0.067-0.022	0.329
Absolute birth weight difference, kg[†]			
Anterior-posterior	Reference	-	-
Posterior-posterior	0.004	-0.032-0.040	0.833
Anterior-anterior	-0.031	-0.065-0.004	0.079

Table S2. The relationship between neonatal outcomes and placental location in a multivariate adjusted model. Note: aOR, adjusted odds ratio; CI, confidence interval; SGA, small for gestational age; * adjusted for neonatal sex, abnormal cord insertion and pre-gestational or gestational diabetes mellitus; # GEE model adjusted for neonatal sex, abnormal cord insertion and pre-gestational or gestational diabetes mellitus; †adjusted for gestational age at delivery, neonatal sex, abnormal cord insertion and pre-gestational or gestational diabetes mellitus;
